# Metal Complexes of Diisopropylthiourea: Synthesis, Characterization and Antibacterial Studies

**DOI:** 10.3390/ijms12107186

**Published:** 2011-10-21

**Authors:** Peter A. Ajibade, Nonkululeko H. Zulu

**Affiliations:** Department of Chemistry, University of Fort Hare, Private Bag X1314, Alice 5700, South Africa; E-Mail: nzulu@ufh.ac.za

**Keywords:** metal complexes, diisopropylthiourea, antibacterial, drug resistance

## Abstract

Co(II), Cu(II), Zn(II) and Fe(III) complexes of diisopropylthiourea have been synthesized and characterized by elemental analyses, molar conductivity, magnetic susceptibility, FTIR and electronic spectroscopy. The compounds are non-electrolytes in solution and spectroscopic data of the complexes are consistent with 4-coordinate geometry for the metal(II) complexes and six coordinate octahedral for Fe(III) complex. The complexes were screened for their antibacterial activities against six bacteria: *Escherichia coli*, *Pseudomonas auriginosa*, *Klebsiella pneumoniae*, *Bacillus cereus*, *Staphylococcus aureus* and *Bacillus pumilus*. The complexes showed varied antibacterial activities and their minimum inhibitory concentrations (MICs) were determined.

## 1. Introduction

Substituted thioureas are known to form stable, neutral coordination compounds with a variety of transition metal ions and some has been structurally characterized [1]. The chemistry of substituted thiourea derivatives has attracted attention because of their potential use as reagents for the separation of metal ions [2] and as antibacterial [3], antiviral [4] and antifungal agents [5]. Apart from their various applications, these ligands are of interest because they have three potential coordination sites: the sulfur of the CS group and the nitrogen atom of the NH groups. Infectious diseases are a major cause of death especially in developing countries [6,7]. The development of antimicrobial drugs, particularly antibiotics, has long being touted as one of the great medical success stories of the twentieth century [8]. At present, resistance against antimicrobial agents is becoming a public health problem worldwide [9,10]. In the search for novel therapy, recent research [11–14] has demonstrated that attaching organic drugs to metal containing fragments can enhance their activity. As part of our studies [15–21] to develop metal based therapeutic agents, we report the synthesis, characterization and antimicrobial screening of metal complexes of diisopropylthiourea.

## 2. Results and Discussion

**Figure 1:**
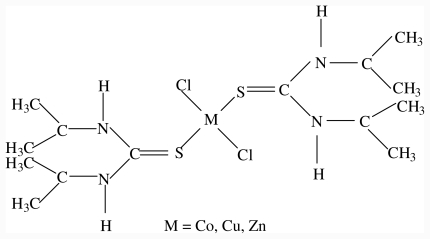
Proposed structure for some M(II) complexes of diisopropylthiourea.

**Figure 2:**
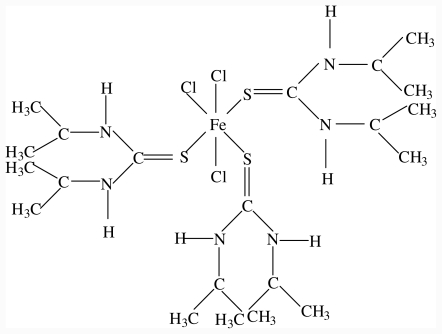
Proposed structure for iron(III) complex of disopropylthiourea.

Cobalt(II), Copper(II), Zinc(II) and iron(III) complexes of diisopropylthiourea has been synthesized by the reaction between the metal salts and diisopropylthiourea in ethanol. The complexes are air stable, non-electrolytes in solution and were characterized by elemental analysis, UV-Vis, FTIR, magnetic susceptibility measurements, conductivity, and M.pt/decomposition temperature. The zinc(II) complex, [ZnCl_2_(diptu)_2_] was further characterized by single crystal X-ray crystallography. The analytical and spectroscopic data for the complexes are presented in Table 1 and their proposed structures in Figures 1 and 2. The Zn(II), Co(II) and Cu(II) complexes formed four coordinate species by coordinating to two molecules of diisopropylthiourea through the sulfur atom and two chloride ions while the Fe(III) complex coordinated with three molecules of diisopropylthiourea and the octahedral geometry around the metal ion was completed by coordinating to three chloride ions.

### 2.1. The Molecular Structure of [ZnCl_2_(diptu)_2_]

**Figure 3:**
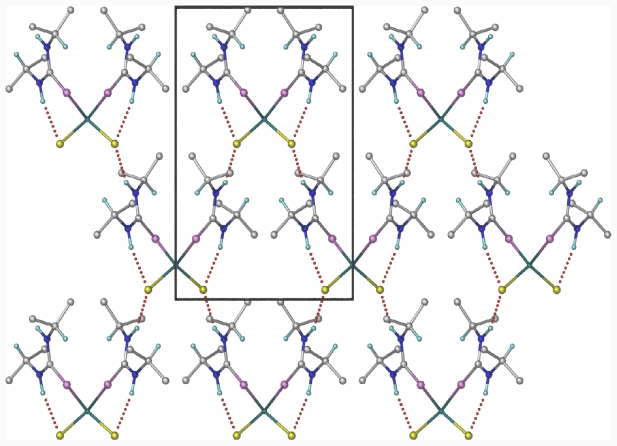
Molecular structure of [ZnCl_2_(diptu)_2_]. The atoms of the asymmetric unit are labeled, the other half of the molecule is generated via symmetry transformation −*x*, *y*, −*z* + 1/2.

Single crystals suitable for X-ray analysis was obtained from methanol/dichloromethane mixtures by slow evaporation at room temperature. The crystals were air stable and moisture stable. The molecular structure of the complex along with atom numbering scheme is given in Figure 3, parameters for crystal data collection and structure refinements are in Table 2. Selected bond lengths and angles are presented in Table 3 and the hydrogen bonding matrix is shown in Table 4. The complex, [ZnCl_2_(diptu)_2_] crystallizes in orthorhombic, space group Pbcn and the geometry around the zinc atom is best described as a distorted tetrahedral [ZnS_2_Cl_2_] environment. The tetrahedral coordination around the zinc atom consists of two monodentate diisopropylthiourea ligands and two chloride ions at the apices (Figure 3). The orientation of the diisopropylthiourea ligands, defined by M = Co, Cu, Zn Cl–Zn–S–C and Zn–S–C–N torsion angles, is determined by intramolecular N–H···Cl hydrogen bonding interaction.

The Cl(1)–Zn(1)–Cl(1)#1, Cl(1)–Zn(1)–S(1), Cl(1)#1–Zn(1)–S(1) bond angles are 112.85(2)°, 110.761(14)° and 106.083(13)°, respectively. These angles deviate from the regular tetrahedral value of 109.47°. These distortions results in secondary deviations of the other perinuclear angles from the ideal value. The deviation from ideal tetrahedral geometry is probably due to the steric hindrance of the 1,3-diisopropylthiourea ligands and the slight difference in electro negativities value of the S and Cl atoms respectively. The bond angles around the zinc atom are in the range 100.64(5)–112.85(2)°. The average C–S and C–N distances [1.7363(14) and 1.4020(18) Å] respectively of the diisopropyl thiourea molecules agree well with the CSD values [1.725(19) and 1.322(16) Å] for terminally coordinated thiourea [22]. In the Zn(II) complex, the C–N and C–S bonds shows intermediate bond length between single and double bond due to the delocalization of electron in the amide bond. The two Zn–Cl bond distances are similar to each other, as are the Zn–S bond distances. The Zn–S bond show no significant differences from the Zn–S bond distances in other known zinc-sulfur complexes in a pseudo-tetrahedral environment [23,24].

**Figure 4:**
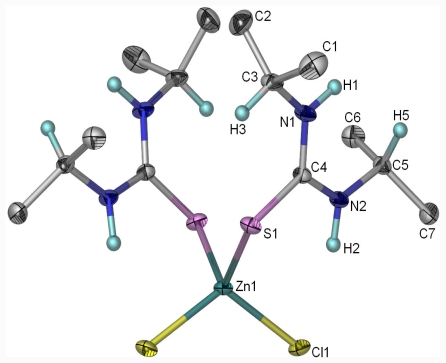
This diagram shows two-dimensional hydrogen bonding network perpendicular to the c axis. All the methyl hydrogen’s are omitted for clarity. The hydrogen bonds are shown as dotted lines.

**Figure 5:**
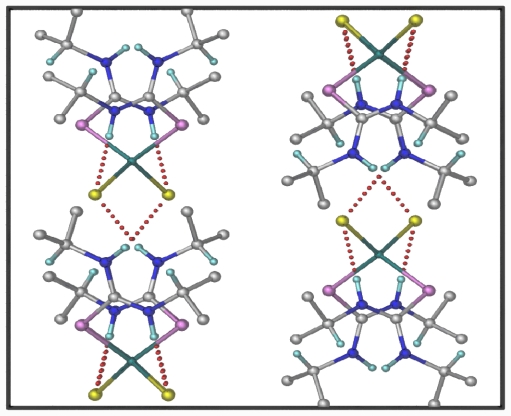
Projection viewed of [ZnCl_2_(diptu)_2_] down [100]. All the methyl hydrogen’s are omitted for clarity. The hydrogen bonds are shown as dotted lines.

The packing of [ZnCl_2_(diptu)_2_)_2_] molecules in the crystal lattice (Figure 4) consists of parallel chains formed by intermolecular H-bonding. Each discrete molecule interacts likewise with four neighboring molecules. For the intermolecular H-bond, each molecule of the ligand acts as an H-bond donor as well as an acceptor. The two dimensional hydrogen bonding network observed in the structure are intermolecular hydrogen bond, viz. N(1)–H(1)…Cl(1)#2, and intramolecular hydrogen bond, viz. N(2)–H(2)…Cl(1). As a result of the intermolecular hydrogen bond, one molecule of the complex is linked to four adjacent molecules (Figures 4 and 5). The NH groups of the diisopropylthiourea ligands are arranged such that they facilitate the formulation of intra- and inter-molecular hydrogen bonds, involving Cl– anions as acceptors in both cases (Table 4). This is similar to what is seen in other zinc(II) bis(thiourea) polymers [25,26]. The intermolecular hydrogen bonds link the molecules into infinite hydrogen bonded networks

### 2.2. Infrared Spectra of Metal Complexes of Diisopropylthiourea

The solid state IR spectra of diisopropylthiourea and the metal complexes in the region 4000–400 cm^−1^ were compared and assigned on careful comparison. In the region 3450–3100 cm^−1^, the N–H vibrations that appear as a broad peak at 3450 cm^−1^ in the spectrum of the ligand shifted to higher wave numbers in the complexes. These shifts might be attributed to the S→M bond and increase in the C–N bonds [21,27,28]. The band at about 1480 cm^−1^ in the ligand shifted to about 1486–1524 cm^−1^ in the spectra of the complexes. These shifts can be ascribed to increase in the double bond character of the C–N bond on complex formation. The infrared spectra of the complexes confirm that the thiourea ligands are coordinated to the metal ions via the exocyclic sulfur atom with a reduction in the π-electron density of the exocyclic C=S bond. In addition, the υ (C=S) bond of the free ligand is red-shifted to lower frequencies in the complexes.

### 2.3. Electronic Spectra of Metal Complexes of Diisopropylthiourea

Co(II) complexes is the most important d^7^ species known in all the coordination numbers and because of its stereochemical diversity, its spectra have been widely studied [29,30]. In a cubic field, three spin allowed transitions are anticipated because of the splitting of the free-ion, ground ^4^F term, and the accompanying ^4^P term. For d^7^ ions in tetrahedral crystal fields, the splitting of the free-ion, ground F term is the reverse of that in octahedral field, so that ^4^A_2g_(F) lies lowest. Thus spectra of cobalt (II) complexes usually consist of a broad, intense band in the visible region with a weaker one in the infrared. The ^4^A_2_(F)→^4^T_1_(F) and ^4^A_2_→^4^T_1_(P) transition appear as multiple absorption in the near and visible regions, respectively. There are also bands occurring as shoulder in the high frequency side assigned to ^4^A_2_(F)→^4^T_1g_(F) [^4^A_2_(F)→^4^T_2_(F)]. Examination of this part of the infrared has sometimes indicated the presence of a band, though overlying vibrating bands makes interpretation difficult. The electronic spectrum of [CoCl_2_(diptu)_2_] showed two bands at 600 and 650 nm typical of Co(II) tetrahedral complexes. A room temperature magnetic moment of 4.0 B.M confirms the proposed tetrahedral geometry for the complex.

[CuCl_2_(diptu)_2_] consists of bands in range 540–650 nm and 360–430 nm and were assigned to ^2^B_1g_→^2^A_1g_ and ^2^B_1g_→^2^B_2g_(p) respectively indicating that the complex is in a square planar environment [31,32]. The complex has a room temperature magnetic moment of 2.10 B.M indicating a mononuclear Cu(II) complex. Generally, mononuclear Cu(II) complexes have magnetic moments of about 1.9–2.2 B.M due to orbital contribution to the magnetic moment and spin orbital contributions [33]. The electronic spectrum of [Fe(diptu)_2_] exhibited charge transfer transitions from 600 to 340 nm. Effective magnetic moment of 5.1 B. M. confirms that the complex is high-spin.

### 2.4. Antibacterial Studies of the Complexes

The complexes were screened against six bacteria microorganisms: *Escherichia coli* (ATCC 8739), *Pseudomonas aeruginosa* (ATCC 7700), *Klebsiella pneumoniae* (ATCC 4352), *Basicullus cereus* (ATCC 10702), *Staphylococcus aureus* (ATCC 6538) and *Bacillus pumilus* (ATCC 14884) typed cultures as obtained from the American Type Culture Collection (ATCC). The zones of inhibition of the disc diameters and minimum inhibition concentrations (MICs) of the metal complexes against the microorganisms tested are shown in Tables 5 and 6. The results obtained from the antibacterial studies revealed *S. aureus* to be the most sensitive microorganism with the largest inhibition zones 14.5 mm and 14 mm for [CoCl_2_(diptu)_2_] and [FeCl_3_(diptu)_3_] complexes respectively and 11.5 mm against [CuCl_2_(diptu)_2_]. The smallest zones of inhibition were exhibited by *P. aureginosa* (9 mm) for [CoCl_2_(diptu)_2_] and 9.5 mm for both [CuCl_2_(diptu)_2_] and [FeCl_3_(diptu)_3_] complexes. The MIC values for the microorganisms vary between 2.5 to 5 mg/mL with the entire microorganism having MIC value of 2.5 mg/mL against [CoCl_2_(diptu)_2_] and 5 mg/mL against [CuCl_2_(diptu)_2_] except for *E. Coli* against [CuCl_2_(diptu)_2_] with MIC value of 2.5 mg/mL. It can thus be concluded that the metal complexes have a broad spectrum antibacterial activity against all the tested microorganisms. Although the antibacterial activity can best be described as mild to moderate, the complexes can be optimized further through derivatization to enhance their antibacterial potential.

## 3. Experimental Section

### 3.1. Materials and Instrumentation

All reagents, metal salts, trimethoprim, pyrimethamine and amodiaquine were used as obtained from Aldrich. Elemental analyses were performed at the Micro-analytical laboratory of the School of Chemistry, The University of Manchester, UK. IR spectra were obtained as KBr discs on a Perkin-Elmer Paragon 1000 FTIR spectrophotometer equipped with CsI window (4000–250 cm^−1^). UV-Vis spectra were obtained on a Perkin-Elmer Lambda 20 spectrophotometer. Magnetic susceptibility measurements were carried out at room temperature using Sherwood scientific magnetic susceptibility balance. Diamagnetic corrections were made using Pascal’s constant [34].

### 3.2. Synthesis of Metal Complexes of Diisopropylthioruea

#### 3.2.1. Synthesis of [CuCl_2_(diptu)_2_]

CuCl_2_·2H_2_O (0.8525 g, 5 mmol) dissolved in 40 mL ethanol was added to a solution of diisopropylthiourea (1.6028 g, 10 mmol) dissolved in 40 mL ethanol and refluxed for 12 h to give yellowish colour solution. The solution was concentrated *in vacuo*, filtered and precipitate washed with ethanol, diethylether and air dried. Yield: 1.3 g (57 %)

#### 3.2.2. Synthesis of [CoCl_2_(diptu)_2_]

CoCl_2_·6H_2_O (2.1414 g, 9 mmol) dissolved in 40 mL ethanol was added to a solution of diisopropylthiourea (2.8851 g, 18 mmol) dissolved in 60 mL ethanol and refluxed for 2 h. The resultant blue solution was concentrated *in vacuo*, filtered and the precipitate washed with ethanol, diethylether and air dried. Yield: 3.4 g (84 %).

#### 3.2.3. Synthesis of [ZnCl_2_(diptu)_2_]

ZnCl_2_ (1.2283 g, 9 mmol) dissolved in 40 mL ethanol was added to a solution of diisopropylthiourea (2.8849 g, 18 mmol) in 40 mL ethanol and refluxed for 6 h. The whitish solution obtained was concentrated *in vacuo*, filtered and the precipitate washed with ethanol, diethylether and air dried. Yield: 3.2 g (79 %).

#### 3.2.4. Synthesis of [FeCl_3_(diptu)_2_]

FeCl_3_·6H_2_O (1.6218 g, 6 mmol) dissolved in 40 mL ethanol was added to a solution of 1,3-diisopropylthiourea (2.8850 g, 18 mmol) in 60 mL ethanol and refluxed for 5 h. The solution was concentrated *in vacuo*, filtered and the precipitate washed with ethanol, diethylether and air dried. Yield: 2.6 g (67 %).

#### 3.2.5. X-ray Crystallography

Single-crystal X-ray diffraction data were collected on a Bruker KAPPA APEX II DUO diffractometer using graphite-monochromated Mo-Kα radiation (χ= 0.71073 Å). The crystal was coated with paratone-N oil and mounted on a cryoloop. Data collection was carried out at 173(2) K to minimize solvent loss, possible structural disorder and thermal motion effects. Temperature was controlled by an Oxford Cryostream cooling system (Oxford Cryostat). Cell refinement and data reduction were performed using the program SAINT [35]. The data were scaled and empirical absorption corrections were performed using SADABS [36]. The structure was solved by direct methods using SHELXS-97 [36] and refined by full-matrix least-squares methods based on F2 using SHELXL-97 and using the graphics interface program X-Seed [37]. The programs X-Seed and POV-Ray [38] were both used to prepare molecular graphic images. All non-hydrogen atoms were refined anisotropically. All hydrogen atoms, except H1 and H2, were positioned geometrically and refined as riding on their parent atoms, with *U*_iso_ (H) = 1.2 *U*_eq_ (C) and 1.5 *U*_eq_ (methyl C). The positions of H1 and H2 were placed in difference Fourier maps and refined independently with simple bond length constraints. The structure was refined successfully with *R* factor 0.0223.

#### 3.2.6. Antibacterial Studies of the Metal Complexes

The antibacterial activity of the metal complexes was evaluated against six bacteria obtained from the American Type Culture Collection (ATCC). The antibacterial activity of the complexes was qualitatively determined by a modified disc diffusion method [23]. Antibacterial activity was indicated by the presence of clear inhibition zones around the discs. Substances showing positive antibacterial activity via the disc diffusion assay were subjected to the broth diffusion method for quantitative measurement of microbiostatic (inhibitory) activity [39]. The lowest concentration that completely inhibited visible microbial growth was recorded as the minimum inhibitory concentration (MIC). Amphicillin was used as positive control for bacterial growth.

## 4. Conclusions

Diisopropylthiourea ligand acts as unidentate ligand through the sulfur atom forming strong complexes with Co(II), Cu(II), Zn(II) and Fe(III). The complexes were characterized by elemental analyses, molar conductivity, magnetic susceptibility, FT-IR and spectroscopic techniques. The single crystal X-ray structure of [ZnCl_2_(diptu)_2_] is also reported. Evidence from the coordination chemistry of the crystal structure of the Zn(II) complex is dominated by strong intramolecular hydrogen bonds which lock the diisopropylthiourea N–H moiety and the chloride ion. There are also intermolecular NH–Cl interactions. The antibacterial studies of three of the complexes were studied and their minimum inhibition concentrations determined.

## Figures and Tables

**Table 1:** Analytical data for metal complexes of diisopropylthiourea.

Compounds	Empirical Formula	Formula Weight	Elemental Analyses (Calcd)			
			C	H	N	S
[CuCl_2_(diptu)_2_]	CuCl_2_C_14_H_32_N_4_S_2_	455.01	36.35 (36.96)	6.65 (7.09)	12.09 (12.31)	14.30 (14.09)
[ZnCl_2_(diptu)_2_]	ZnCl_2_C_14_H_32_N_4_S_2_	456.84	37.42 (36.81)	7.22 (7.06)	12.81 (12.26)	13.95 (14.04)
[CoCl_2_(diptu)_2_]	CoCl_2_C_14_H_32_N_4_S_2_	450.39	37.34 (37.33)	6.80 (7.16)	11.92 (12.14)	12.08 (14.24)
[FeCl_3_(diptu)_2_]	FeCl_3_C_21_H_48_N_6_S_3_	643.04	39.75 (39.22)	7.66 (7.52)	13.36 (13.07)	14.81 (14.96)

**Table 2:** Summary of crystal data and structure refinement for [ZnCl_2_(diptu)_2_].

Compound	[ZnCl_2_(diptu)_2_]
Empirical formula	C_14_H_32_Cl_2_N_4_S_2_Zn
Formula weight	456.83
Temperature, K	173(2)
Wavelength, Å	0.71073
Crystal system	Orthorhombic
Space group	Pbcn

**Unit cell dimensions**	

a (Å)	9.5637(5)
b (Å)	14.8509(8)
c (Å)	15.6554(9)
β (°)	90
γ (°)	90
Volume (A^3^)	2223.5(2)
Z	4
D_calc_ Mg/m^3^	1.365
Absorption coefficient (mm^−1^)	1.536
F(000)	960
Crystal size (mm)	0.11 × 0.09 × 0.05
Theta range (°)	2.53 to 28.31
Limiting indices	−12 <= *h* <= 12, −19 <= *k* = 19, −20 <= *l* <= 20
Reflections collected	16637/2768
Independent reflection	[R(int) = 0.0278
Refinement method	Full-matrix least squares on F2
Completeness to θ = 28.28	100
Data/restraints/parameters/	2768/2/117
Goodness-of-fit on F^2^	1.035
Final R indices [I > 2 sigma(I)]	R1 = 0.0223, wR2 = 0.0516
R indices (all data)	R1 = 0.0313, wR2 = 0.0552
Largest diff. Peak and hole e. Å^−3^	0.324 and −0.206

**Table 3:** Selected bond length (Å) and bond angles (°) for [ZnCl_2_(diptu)_2_].

Bond Length		Bond Angles	
Zn(1)–Cl(1)	2.2634(4)	Cl(1)–Zn(1)–Cl(1)#1	112.85(2)
Zn(1)–Cl(1)#1	2.2634(4)	Cl(1)–Zn(1)–S(1)	110.761(14)
Zn(1)–S(1)	2.3480(4)	Cl(1)#1–Zn(1)–S(1)	106.083(13)
Zn(1)–S(1)#1	2.3480(4)	Cl(1)–Zn(1)–S(1)#1	106.083(13)
S(1)–C(4)	1.7363(4)	Cl(1)#1–Zn(1)–S(1)#1	110.761(14)
N(1)–C(4)	1.3261(18)	S(1)–Zn(1)–S(1)#1	110.37(2)
N(1)–C(3)	1.4779(18)	C(4)–S(1)–Zn(1)	100.37(5)
N(2)–C(4)	1.3328(18)	C(4)–N(1)–C(3)	125.52(12)
N(2)–H(2)	0.961(5)	C(4)–N(1)–H(1)	117.7(11)
N(2)–C(5)	1.4715(18)	C(3)–N(1)–H(1)	116.5(11)
		C(4)–N(2)–C(5)	128.08(12)
		C(4)–N(2)–H(2)	116.3(11)
		N(1)–C(4)–N(2)	120.37(13)
		N(1)–C(4)–S(1)	120.95(11)
		N(2)–C(4)–S(1)	118.67(10)

**Table 4:** Distances (Å) and angles (°) involving hydrogen bonding of [ZnCl_2_(diptu)_2_].

D-H…A	D(D-H)	d(H…A)	d(D…A)	<(DHA)
N(1)–H(1)…Cl(1)#2	0.963(5)	2.476(8)	3.3899(13)	158.5(15)
N(2)–H(2)…Cl(1)	0.961(5)	2.412(6)	3.3580(12)	168.0(16)

Symmetry transformations used to generate equivalent atoms: #1: −*x*, *y*, −*z* + 1/2; #2: −*x* + 1/2, *y* + 1/2, *z*.

**Table 5:** Zones of inhibition of the complexes at 10 mg/mL.

Complex/Bacteria	[CuCl_2_(diptu)_2_]	[CoCl_2_(diptu)_2_]	[FeCl_3_(diptu)_3_]
*E. coli*	12.5	11.5	13.0
*P. auruginosa*	9.5	9.0	9.5
*K. pnemoniae*	10.5	11.0	10.5
*B. cereus*	11.5	13.5	13.0
*S. aureus*	11.5	14.5	14.0
*B. pumilus*	11.5	13.5	12.5

**Table 6:** Minimum inhibition concentrations (MIC) in mg/mL of the metal complexes.

Complex/Bacteria	[CuCl_2_(diptu)_2_]	[CoCl_2_(diptu)_2_]	[FeCl_3_(diptu)_3_]
*E. coli*	2.5	2.5	2.5
*P. auruginosa*	5.0	2.5	5.0
*K. pnemoniae*	5.0	2.5	2.5
*B. cereus*	5.0	2.5	5.0
*S. aureus*	5.0	2.5	2.5
*B. pumilus*	5.0	2.5	2.5
